# *Vibrionaceae* core, shell and cloud genes are non-randomly distributed on Chr 1: An hypothesis that links the genomic location of genes with their intracellular placement

**DOI:** 10.1186/s12864-020-07117-5

**Published:** 2020-10-06

**Authors:** Cecilie Bækkedal Sonnenberg, Tim Kahlke, Peik Haugen

**Affiliations:** 1 Department of Chemistry and Center for Bioinformatics (SfB), Faculty of Science and Technology, UiT The Arctic University of Norway, N-9037 Tromsø, Norway; 2 Climate Change Cluster, University of Technology Sydney, Sydney, NSW Australia

**Keywords:** Pangenome, Genome architecture, *Vibrionaceae*, *Aliivibrio salmonicida*, *Vibrio natriegens*, Gene dosage

## Abstract

**Background:**

The genome of *Vibrionaceae* bacteria, which consists of two circular chromosomes, is replicated in a highly ordered fashion. In fast-growing bacteria, multifork replication results in higher gene copy numbers and increased expression of genes located close to the origin of replication of Chr 1 (*ori1*). This is believed to be a growth optimization strategy to satisfy the high demand of essential growth factors during fast growth. The relationship between *ori1*-proximate growth-related genes and gene expression during fast growth has been investigated by many researchers. However, it remains unclear which other gene categories that are present close to *ori1* and if expression of all *ori1*-proximate genes is increased during fast growth, or if expression is selectively elevated for certain gene categories.

**Results:**

We calculated the pangenome of all complete genomes from the *Vibrionaceae* family and mapped the four pangene categories, core, softcore, shell and cloud, to their chromosomal positions. This revealed that core and softcore genes were found heavily biased towards *ori1*, while shell genes were overrepresented at the opposite part of Chr 1 (i.e., close to *ter1*). RNA-seq of *Aliivibrio salmonicida* and *Vibrio natriegens* showed global gene expression patterns that consistently correlated with chromosomal distance to *ori1*. Despite a biased gene distribution pattern, all pangene categories contributed to a skewed expression pattern at fast-growing conditions, whereas at slow-growing conditions, softcore, shell and cloud genes were responsible for elevated expression.

**Conclusion:**

The pangene categories were non-randomly organized on Chr 1, with an overrepresentation of core and softcore genes around *ori1*, and overrepresentation of shell and cloud genes around *ter1.* Furthermore, we mapped our gene distribution data on to the intracellular positioning of chromatin described for *V. cholerae,* and found that core/softcore and shell/cloud genes appear enriched at two spatially separated intracellular regions. Based on these observations, we hypothesize that there is a link between the genomic location of genes and their cellular placement.

## Background

Bacteria that belong to the family *Vibrionaceae* are rich in most aqueous habitats, from the deep seas to fresh and brackish waters, and in temperature zones ranging from the polar to tropical areas. They exist as free-swimming cells or associated with other organisms, either in a symbiotic relationship or as pathogens of e.g. fish, corals and even humans [1, 2]. Despite the notorious reputation of some *Vibrionaceae* species, (e.g., *Vibrio cholerae* and *Vibrio vulnificus)* it is the diversity of non-pathogenic *Vibrionaceae* species that makes these bacteria so successful and ecologically important [3]. The facultative anaerobic bacterium *Vibrio natriegens,* for example, fixes atmospheric nitrogen (N_2_) into ammonia (NH_3_), and thus provides its surroundings with a critical nutrient [4].

As of April 2020, the RefSeq database contains 306 complete *Vibrionaceae* genomes (representing 57 species), with genomes from new species being added on a regular basis. One characteristic feature shared by almost all *Vibrionaceae* genomes is a highly unusual bipartite structure consisting of a large (Chr 1) and a smaller (Chr 2) chromosome [5, 6]. It is proposed that bacteria with bipartite genomes have a selective advantage for the adaptation to very different environmental conditions [7], and that division into multiple smaller replicons may reduce replication time, thus allowing for faster generation time and a competitive advantage [8, 9]. The unconventional genome constellation is expected to require tightly regulated and synchronized replication to ensure proliferation and control of gene expression during changes in the surrounding environment.

In *V. cholerae,* replication of Chr 1 and Chr 2 is highly coordinated [10]. When the replication fork approaches *crtS* in Chr 1 (Chr 2 replication triggering site), a hitherto unknown mechanism triggers replication of Chr 2 [11, 12]. Interestingly, there is a short pause (corresponding to replication of approx. 200 kbp) between the *crtS* replication and the initiation of Chr 2 replication. The exact function of this pause is yet unknown, but it is hypothesized to be needed for activation of the *rctB* (Chr 2’s own replication initiator) and *ori2* initiation system [12]. In other words, the chromosomal position of *crtS* and the pause contribute to synchronize termination of Chr 1 and Chr 2 replication. Furthermore, the synchronized termination is likely linked to coordination of chromosome segregation and cell division [12].

Another intriguing phenomenon regarding replication of *Vibrio* genomes is that genes surrounding *ori* can be found in multiple copies during the replication process due to successive initiations of replication from *ori* (i.e., multifork replication) [13, 14]. This phenomenon is a hallmark of fast-growing bacteria, such as *V. cholerae* and *V. natriegens*, and is believed to be a growth optimization strategy to satisfy the high demand of essential growth factors during fast growth [15–17]. Using an elegant genetic approach, Soler-Bistué et al. (2015) showed that by relocating the major ribosomal protein gene locus (*s10-spec-α*) of *V. cholerae* further away from *ori1*, growth rate, the gene copy number and mRNA abundance of this cluster were reduced [18]. The authors concluded that there is a strong correlation between chromosomal gene position and effects on the bacterial physiology. Later, the same model system (i.e., *V. cholerae* with relocated *s10-spec-α* locus) was used to study effects on bacterial fitness under slow growth conditions (i.e., no multifork replication) [19]. One conclusion from this study was that bacterial fitness was reduced when the *s10-spec-α* locus was located distal to *ori1*, which demonstrates that genomic positioning of ribosomal protein genes not only affects growth, but also cell fitness across the whole life cycle. In a recent study, Soler-Bistué et al. (2020) showed that relocation of the *s10-spec-α* locus lead to higher cytoplasm fluidity and the authors suggested that changes in the macromolecular crowding of the cytoplasm impacts the cellular physiology of *V. cholerae.* Interestingly, the protein production capacity in *V. cholerae* was independent of the position of the *s10-spec-α* locus [20].

In an interesting approach, Dryselius et al. (2008) used qPCR and microarray to study how copy numbers of genes vary across the entire genome of several *Vibrio* species (*V. parahaemolyticus, V. cholerae* and *V. vulnificus*) under different growth conditions, and then monitored how the data correlated with gene expression levels (also using microarray) [21]. The authors found greatest differences in gene copy numbers across Chr 1 compared to Chr 2 when grown in a rich medium. In general, the trend is that gene copy numbers increase from the terminus towards the origin of replication, and that this increase is reflected by increasing gene expression levels. The same trend was not found for slow-growing bacteria (i.e., when grown in minimal medium). Also, for Chr 2 gene expression levels were low and apparently independent of gene copy number effect. Similar findings were later described in *V. splendidus* [22]. Here, genes located on Chr 1 were 3.6 × more expressed compared to those located on Chr 2, and the highest expression values were typically associated with genes surrounding the origin of replication on Chr 1.

In summary, the genome of *Vibrionaceae* bacteria, which consists of two circular chromosomes, is replicated in a highly ordered fashion. In fast-growing bacteria, replication results in higher gene copy numbers, and increased expression of genes located close to the origin of replication of Chr 1. That the expression of growth-related genes located close to *ori1* is elevated during fast growth is known, but a general picture of which gene types are found close to *ori1,* and how expression of each gene type is affected, is however not known. To address this knowledge gap we revisited the intriguing topic of genome architecture in *Vibrionaceae*. In a pangenome approach we used available genomes to calculate and divide clusters of orthologous genes into the main categories “core”, “softcore”, “shell” (accessory) and “cloud” (unique), and used this information to determine how the corresponding genes are distributed on Chr 1 and Chr 2 of selected *Vibrionaceae* genomes. Data from publicly available gene expression experiments was mapped back to the pangenes to determine gene expression profiles under different environmental conditions such as expression data from the fast-growing bacterium *V. natriegens* grown under optimal or minimal growth conditions, and data from the fish-pathogen *Aliivibrio salmonicida* grown under salt concentration and temperature that mimics the physiological conditions during infection. Our results show a non-random distribution of genes on the two chromosomes of *Vibrionaceae*. The gene distribution was then compared with global gene expression trends, and we find a strong correlation between expression levels and distance from *ori1.* Surprisingly, despite a biased gene distribution pattern, all pangene categories contribute to a skewed expression pattern at fast-growing conditions. Finally, based on our data we propose an hypothesis that describes how pangenes are spatially distributed inside *Vibrionaceae* bacterial cells, and we discuss possible implications of the proposed hypothesis.

## Results

### Pangenome calculations based on 124 complete *Vibrionaceae* genomes identifies 710 clusters of orthologous core genes

To categorize all genes associated with *Vibrionaceae* genomes into distinct classes, we downloaded all complete genomes from the NCBI RefSeq database (124 as of May 2018, see Additional file 1), and then used GET_HOMOLOGUES v3.1.0 [23] to cluster orthologous protein sequences based on the OrthoMCL algorithm. The pangenome calculations identified a total of 61,512 clusters, of which 710 were encoded by genes found in all 124 genomes (i.e., core genes). The remaining clusters are distributed among softcore (encoded by ≥117 genomes), shell (encoded by 116 ≤ and ≥ 3 genomes) and cloud (encoded by ≤2 genomes), and contain 1796, 14,642 and 45,074 clusters, which represents 3, 23 and 73% of the total clusters, respectively. In individual genomes, core gene clusters represent 1.2% of the pangenome, and comprise 10—17% of the total genes. Similarly, softcore constitutes 24—34% (1489—1796 genes per genome) of the total genes.

### Core and softcore genes densely populate the upper half of Chr 1

**Fig. 1:**
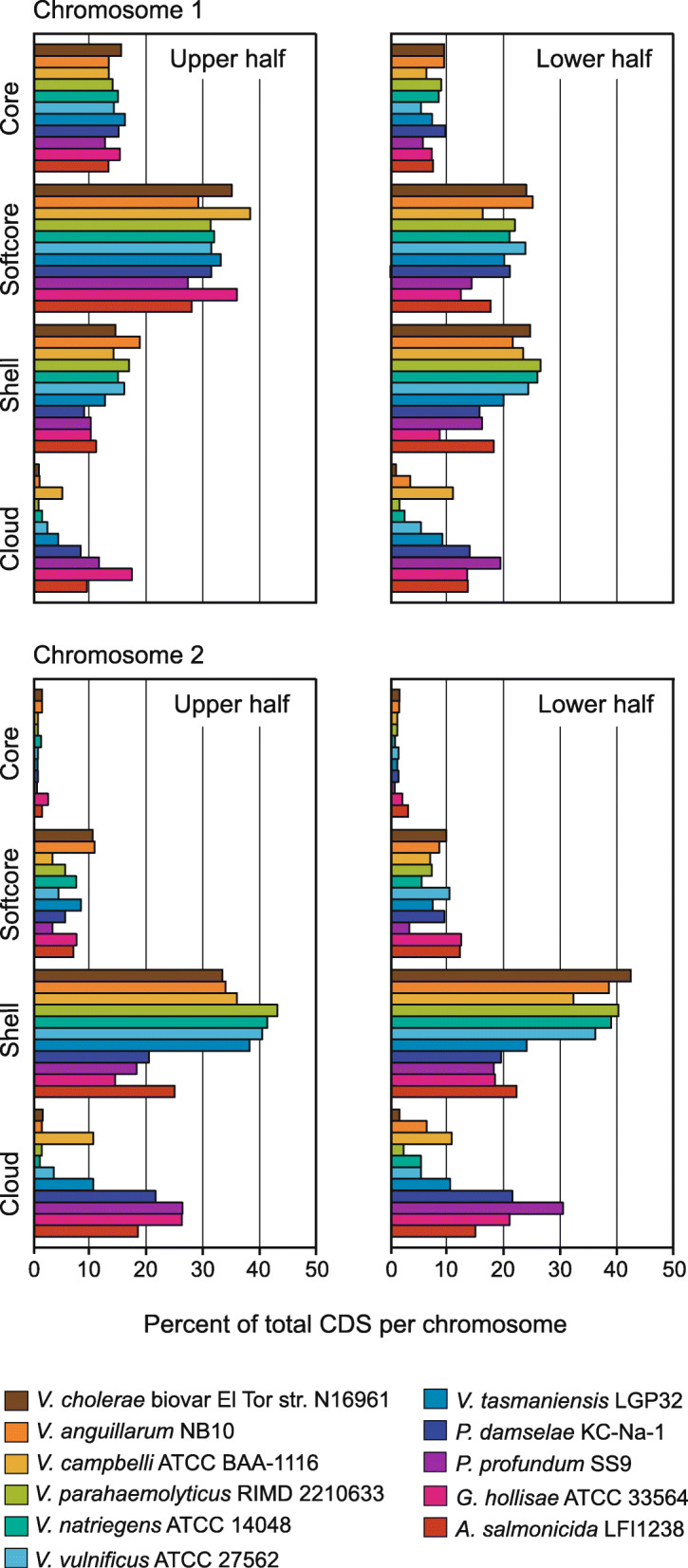
Distribution of the four pangene categories between upper and lower half of 11 *Vibrionaceae* genomes. Bars in the histogram show percent of total CDSs per chromosome for each pangene category. Core and softcore genes are overrepresented on the upper half of Chr 1, shell and cloud genes are overrepresented on the lower half. On Chr 2 the genes are more evenly distributed between the upper and lower halves of Chr 2

The four gene categories core, softcore, shell and cloud, were next mapped to their chromosomal locations to investigate whether they are randomly or non-randomly distributed on each chromosome. First, genes of eleven selected *Vibrionaceae* representatives (see Additional file 2 for phylogeny of the 11 genomes) were classified as either upper or lower (i.e., upper or lower half of the chromosome) based on their chromosomal location on Chr 1 and Chr 2 in relation to their distance of the origin of replication. As presented in Fig. 1 (complete table of pangene distribution is available as Additional file 3 and chi-squared test is available as Additional file 4), core and softcore genes are significantly overrepresented (adjusted chi-square *P*-value ≤0.05) in the upper half of Chr 1 in all investigated genomes. Similarly, shell and cloud genes on Chr 1 are significantly overrepresented (adjusted chi-square P-value ≤0.05) in the lower half of Chr 1 in 8 genomes, thus supporting a non-random distribution of genes on Chr 1. In contrast to Chr 1, genes of all categories are much more evenly distributed on Chr 2. Although shell, cloud and softcore genes show non-random distribution on Chr 2 in some of the investigated genomes (softcore 3/11, shell 1/11, cloud 2/11), the majority of genomes show no significant bias (adjusted chi-square *P*-value ≤0.05). Furthermore, core genes were not significantly overrepresented in either lower or upper half of Chr 2 in any of the genomes.

**Fig. 2:**
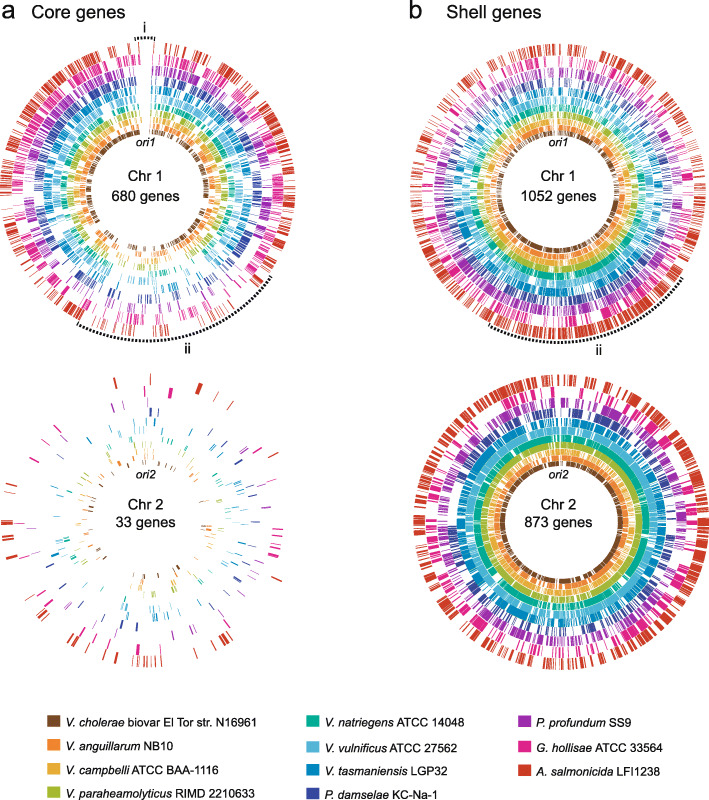
Distribution of 710 core genes in 11 *Vibrio* genomes. Location of core (**a**) and shell (**a**) genes on Chr 1 and Chr 2 of 11 *Vibrionaceae* genomes. Circular plots are arranged regarding the phylogenetic relationship of the investigated isolates. Each plot is centered at a gene assumed to be close to the replication origin: *mioC* on Chr 1 and *rtcB* on Chr 2. As shown, a majority of core genes on Chr 1 is located closer to *ori1* than to ter. Shell genes show the opposite distribution pattern on Chr 1, where majority of shell genes accumulate closer to ter. On Chr 2 both core and shell genes are randomly distributed. The dashed line “i” indicates a region on Chr 1 surrounding *ori1* that contains very few core genes. The dashed line “ii” shows a region on Chr 1 of approximately 500 kb surrounding ter that is more sparsely populated with core genes than the rest of the chromosome

To provide a more fine-grained picture of the core (710—721) and shell (749—2753) gene distributions, we plotted the distribution of core and shell genes on Chr 1 and Chr 2 of eleven *Vibrionaceae taxa* using the genome comparison tool Circos [24] (Fig. 2). Each plot was centered on *mioC* (Chr 1) and *rctB* (Chr 2). Our results show that although the exact distribution pattern varies between species, the biased distributions of core and shell, as described above, are striking and readily visible with the naked eye. Interestingly, although core genes densely populate the upper half of Chr 1, the region immediately surrounding *ori1* contains very few core genes. This region (denoted “i” in Fig. 2) is, in contrast, densely populated by softcore genes (at least in *V. natriegens* and *A. salmonicida,* see section below). Also, a region (denoted “ii” in Fig. 2) of approximately 500 kb surrounding *ter1* is densely populated with shell genes (and hence sparsely populated with core genes). For Chr 2, the chi-square test supported no significant bias in gene distribution (Additional file 4), and Fig. 2b supports this general picture although some local clustering of gene categories will occur. In summary, the results presented here reveal that core, softcore, shell and cloud genes are non-randomly distributed on Chr 1. Core and softcore genes are more likely to be located on the upper half of Chr 1, whereas shell and cloud genes tend to be located closer to the replication terminator. For Chr 2, the distribution of the four pangene categories are in general randomly distributed showing locational bias only for a few genomes.

### Expression levels of genes located on Chr 1 of *V. natriegens* and *A. salmonicida* generally correlate with distance to *ori1*

**Fig. 3:**
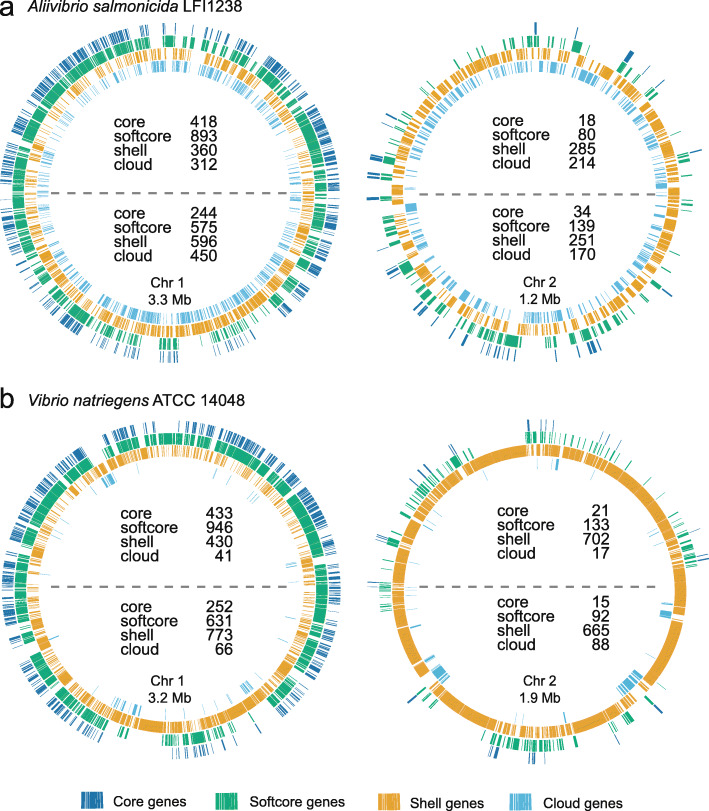
Distribution of the four pangene categories on Chr 1 and Chr 2 for (**a**) *A. salmonicida* LFI1238 and (**b**) *V. natriegens* ATCC 14048. The number of genes in each pangene category in the upper and lower half is written inside each chromosome. A dashed line visualises the separation of the upper and lower half of the chromosomes

Figure 3 shows how core, softcore, shell and cloud pangenes are distributed on Chr 1 and Chr 2 of *V. natriegens* and *A. salmonicida.* The pattern is consistent with the biased gene distribution pattern described above, with core and softcore genes being overrepresented at the upper half of Chr 1, and shell and cloud genes being overrepresented at the lower half. The two species were chosen as models for comparison of gene expression data with pangene distribution patterns. Specifically, we were curious to examine if regions that are densely populated by core/softcore pangenes are expressed at high levels, compared to regions more sparsely populated by core/softcore pangenes. This expectation is based on previous data from *V. parahaemolyticus* and *V. cholerae,* which showed that growth rates have large impacts on the copy number (gene dosage) of genes located on Chr 1, as well as on gene expression levels [9, 10, 21]. Fast- and slow-growing bacterial representatives were therefore chosen for this particular comparative analysis. *V. natriegens* is a fast-growing bacterium commonly found in estuarine mud, with doubling times below 10 min at favourable conditions [25]. *A. salmonicida* is, in contrast, a slow growing *Vibrionaceae* bacterium, and the causative agent of cold-water vibriosis in e.g., Atlantic salmon and cod [26, 27]. To correlate gene distribution with gene expression data, publicly available RNA-seq data of *V.* natriegens and *A. salmonicida* were downloaded from the Sequence Read Archive [28] at NCBI. For *V. natriegens,* datasets from growth in minimal and optimal (rich) medium at 37 °C to mid log phase were chosen [29]. For *A. salmonicida*, a dataset originating from growth in LB medium containing 1% NaCl at 8 °C to mid log phase was used [30]. EDGE-PRO 1.3.1 [31] was used to align cDNA reads to the *V. natriegens* ATCC 14048 (NBRC 15636, DSM 759) (assembly no. GCA_001456255.1) or *A. salmonicida* LFI1238 (assembly no. GCF_000196495.1) genome, and to calculate expression values as reads per kilobase per million (RPKM) for all protein coding sequences (CDS).

**Fig. 4:**
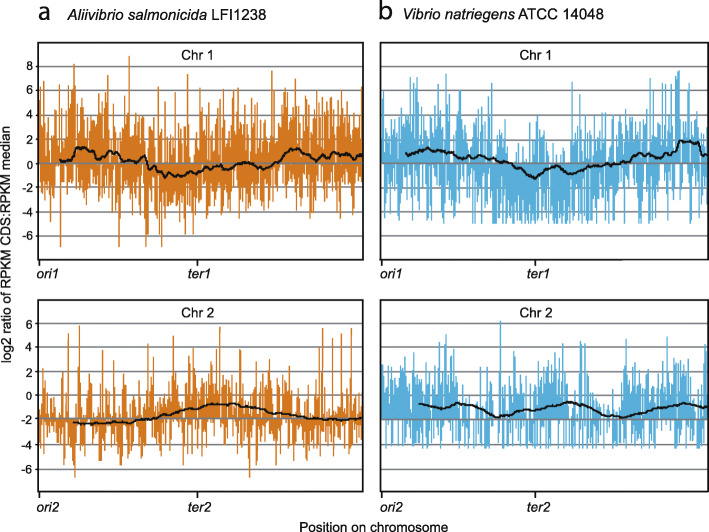
Global expression maps of (**a**) *A. salmonicida* LFI1238 and (**b**) *V. natriegens* ATCC 14048 chromosomal genes centered around the median. Data points (log_2_ ratio RPKM CDS:RPKM median) for each CDS are shown, as well as a trend line averaged over a sliding window of 200 data points. *V. natriegens* ATCC 14048 is grown under fast-growing conditions and *A. salmonicida* LFI1238 is grown under suboptimal conditions

Figure 4 shows global expression maps of *V. natriegens* and *A. salmonicida* chromosomal genes centered around the median. Data points (log_2_ ratio RPKM CDS:RPKM median) for each CDS are shown, as well as a trend line averaged over a sliding window of 200 data points. For Chr 1 the general picture is similar in all three datasets, i.e., RPKM values are typically above the median value at the upper half (i.e., the region closest to the origin of replication), but lower at the region surrounding the terminus, independent of growth conditions. This is somewhat surprising since the observed expression patterns described above was expected for fast growing cultures (i.,e *V. natriegens* in rich medium), but not for slow growing cultures (i.e., *A. salmonicida* in LB 1% NaCl and 8 °C and *V. natriegens* in minimal medium, see Additional file 5). The rationale is that gene copy numbers (also known as “gene dosage”), and thus expression levels are expected to be correlated with growth rates/multifork replication [21].

A more detailed circular expression map is available in Additional file 6 and shows that region “i” (see Fig. 2), which encodes mostly softcore genes, contains a highly expressed proton-translocating ATP synthase (F_0_F_1_ class) gene cluster (atpIBEFHAGDC). The ATPase cluster is well described in *Escherichia coli* as an operon located 84 min on the chromosome (close to *oriC*), and with gene expression levels varying according to cell growth rate [32]. The ATP synthase cluster represents softcore genes, and are present in both bacteria. Moreover, the detailed map shows that region “ii”, which is densely populated with shell genes, differs from the remaining lower half of Chr 1 by being expressed far below median in *V. natriegens* at both fast and slow growth conditions. For *A. salmonicida* the main picture is the same, but less pronounced, meaning that the majority of shell genes located in “ii” are expressed below median.

For Chr 2, the results are more ambiguous, although overall similar between minimal and rich growth. For *A. salmonicida,* expression around the terminus is, on average, higher compared to that of regions adjacent to *ori2*. For *V. natriegens*, expression is generally higher than median in regions surrounding the terminus, but varies across the remaining parts of Chr 2. Similar to Chr 1, little difference could be determined between the slow- and the fast-growing datasets of Chr 2.

In summary, we found that global expression levels for Chr 1, consistently correlate with the distance to the origin of replication. The log2 ratio of RPKM CDS:RPKM median decreases as the distance from origin of replication increases.

### All pangene categories contribute to higher expression levels around *ori1* at fast-growth conditions, but not at slow-growth conditions

The global trend described above can be explained by generally higher expression levels of all pangene categories located close to *ori1*, or, higher expression of three or less of the four pangene categories. To discriminate between the two alternatives, we calculated the RPKM median value for each pangene category, and compared the median values for genes located on the upper or lower halves of Chr 1 (Table 1). The Wilcoxon signed-rank test strongly support (P-adj ≤ 0.05) that median values for all four pangene categories are significantly higher for genes located on the the upper half, i.e., when *V. natriegens* is cultured at fast-growth (“optimal”) conditions. Notably, when grown under slow-growing conditions, median values for softcore, shell and cloud genes located on the upper half are significantly higher. Core genes are in contrast, expressed at equal levels on both halves. This applies for both *V. natriegens* (RPKM median = 370 and 360, *P*-adj = 0.321) in minimal medium, and *A. salmonicida* (RPKM median = 301 and 309, *P*-adj = 0.717) at suboptimal conditions. Conversely, we can therefore state that genes from all pangene categories located on the lower half are generally expressed at lower levels compared to those on the upper half (except for core genes at slow growth conditions). To summarize, we conclude that gene expression levels correlate with distance to *ori1* (Fig. 4), and genes from all four pangene categories contribute to this trend when grown under fast-growing conditions, whereas softcore, shell and cloud genes contribute at slow-growing conditions.

**Table 1:** Comparison of gene expression levels for pangenes located on the upper or lower halves of Chr 1

	***A. salmonicida***				***V. natriegens*** slow-growth				***V. natriegens*** fast-growth			
	core	softcore	shell	cloud	core	softcore	shell	cloud	core	softcore	shell	cloud
Upper half^a^												
Q_1_	152	118	42	42	188	126	21	5	249	170	36	37
Q_2_	301	245	89	67	370	288	71	147	447	341	93	269
Q_3_	853	633	197	197	1101	760	190	426	1059	719	241	581
Max	34,254	34,254	6473	13,656	23,238	23,238	17,161	5533	35,274	35,274	28,737	4049
Lower half^a^												
Q_1_	151	89	34	25	143	83	4	4	178	109	0	0
Q_2_	309	207	65	47	360	192	28	18	328	232	26	17
Q_3_	695	486	133	82	966	565	74	59	696	480	97	62
Max	53,501	8098	19,837	23,646	14,116	14,116	15,800	463	16,521	17,549	17,550	535
*P*-value Q_2_^b^	0.71	0.01	0.00	0.00	0.32	0.00	0.00	0.00	0.00	0.00	0.00	0.00

^a^ Q_1_ is the RPKM value at the first quartile. Q_1_ is defined as the middle number between the smallest number and the median (i.e., the second quartile Q_2_), if the data numbers (in this case RPKM values) are ordered from smallest to largest. The third quartile (Q_3_) is the middle value between the median (Q_2_) and the maximum (Max) value

^b^ Adjusted *P*-values from Wilcoxon signed-rank test, to test if Q_2_ values (median) of genes located on the upper half of Chr 1 are significantly different from Q_2_ values of genes located on the lower half. Values below 0.05 are considered significant

## Discussion

Inspired by the discovery of multifork replication and increased copy numbers of genes surrounding the origin of replication, researchers have for decades studied how different categories of genes are distributed on chromosomes and at which level these genes are expressed. Here, we revisited this topic and describe hitherto hidden/unrecognized global gene distribution and expression patterns in *Vibrionaceae*. First, we mapped pangenes to their chromosomal positions and revealed that core and softcore genes are found heavily biased towards the *ori1* of Chr 1. Shell genes are, in contrast, overrepresented at the opposite part of Chr 1 (i.e., close to *ter*). We next found that gene expression strongly correlates with chromosomal distance to *ori1*. This trend is caused by higher expression of all pangene categories at fast-growing conditions, whereas softcore, shell and cloud genes are responsible for biased (higher) expressing on the upper half of Chr 1 at slow-growing conditions.

### Pangene categories are non-randomly distributed on Chr 1

In this work we report a clear pattern where core/softcore genes are overrepresented on the upper half of Chr 1 of *Vibrionaceae*, particularly at regions corresponding to 10–11 and 1–2 O’clock on Chr 1, and shell/cloud genes are overrepresented in the *ter1* region (Fig. 2). In comparison, no clear pattern was recorded for Chr 2, i.e., the distribution of pangenes appear generally independent of location. For Chr 1, the core/softcore gene distribution pattern resembles that described for genes involved in translation and transcription in *E. coli* [16, 17, 33] and in several *Vibrio* species [16, 17, 21]. More precisely, Couturier and Rocha (2006) showed that genes involved in translation and transcription in four *Vibrio* species are typically found close to *ori1* of Chr 1. Chr 2 contained, in contrast, fewer genes related to translation and transcription than would be expected. Iida and coworkers [21] later found that genes related to growth (both essential and contributing) are located in close proximity to *ori1* in *V. cholerae*. Overrepresentation of core/softcore genes, many of which are important for growth, at the region proximate to *ori1* of *Vibrionaceae* Chr 1 can be explained by an increase in demand for *ori1*-proximate gene products during fast growth (i.e., multifork replication results in elevated gene copy numbers and increased transcription levels). For example, genes that encode ribosomal RNA and ribosomal proteins are found clustered in the upper half of Chr 1, and are expressed at extremely high levels, which support this hypothesis.

Moreover, we found that during fast growth of *V. natriegens,* core, softcore, shell and cloud genes are all expressed at higher levels on the upper half of the chromosome compared to the lower half. In slow-growing *V. natriegens* and *A. salmonicida*, only softcore, shell and cloud genes followed the same trend, which suggests that regulatory mechanisms other than “gene dosage” are in play, to ensure a relatively low and uniform expression of core genes independent of chromosomal position during slow growth.

### Why are core and softcore genes clustered at the old pole area of cells?

**Fig. 5:**
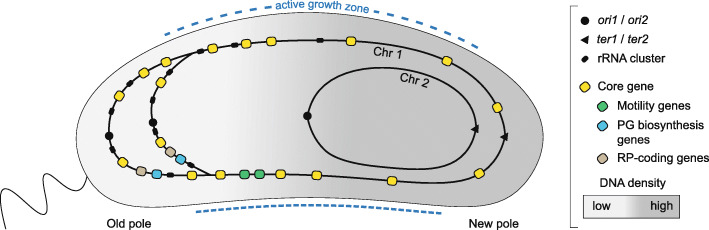
Subcellular distribution of Chr 1 and Chr 2 in *V. cholerae*. Core genes are spatially enriched in the intracellular region near the old pole. Coloured core gene clusters (related to motility, peptidoglycan biosynthesis and ribosomal proteins) represent core gene products that co-localize with growth/survival-related reactions in the old pole of the cell. Two replication origins on Chr 1 indicate multifork replication. Active growth zones are indicated with blue dashed lines along the axis of the cell. Small dashes illustrate fast peptidoglycan growth and long dashes illustrate slower growth

It is well documented in the literature that the intracellular space of bacteria is highly organized, with defined structures at specific locations (reviewed by Surovtsev and Jacobs-wagner 2019) [34]. For example, Chr 1 and Chr 2 of *V. cholerae* are spatially organised in a longitudinal orientation inside the cell*,* with their chromatin stretching from one pole to the other. *ori1* and *ter1* of Chr 1 are located at the old and new poles, respectively, whereas *ori2* and *ter2* of Chr 2 stretches from the new pole towards the cell’s center, respectively (Fig. 5). The organization of Chr 1 and Chr 2 in *V. cholerae* has been established by both fluorescence tag microscopy [9, 35, 36] and chromosome conformation capture (3C) [11]. In the light of this knowledge, our data then suggest that core/softcore and shell/cloud genes are enriched at two spatially separated intracellular regions, i.e., at the two extreme poles of *Vibrionaceae* cells, given that the spatial positioning of chromatin described for *V. cholerae* applies to all representatives within the family. We emphasize, however, that this hypothesis is based on limited data and should be further tested in future experiments before any strong conclusion can be made. Below we further speculate on why core and softcore genes appear clustered at the old (flagellated) pole area.

Given a non-random structural organization of the genes (as hypothesized above), this then suggests to us that there is a link between gene placement and their function, and that the underlying reasons for the strong distribution pattern could be very complex. The full complexity of factors that affects gene expression can be illustrated by e.g., chromatin packing [37–41], nucleoid-associated proteins (NAPs) [42–44], Structural Maintenance of Chromosome complex (SMC) [45], RNA polymerase (RNAP) [46–50], transcription factors and promoter strength/chromosomal position [43, 51] and macromolecular crowding [20]. Perhaps the most fundamental factor is chromatin packing and organization. The density of chromatin is determined by a number of circumstances, including differential abundance/availability of macromolecular machineries [38, 41, 46–50, 52, 53]. In this respect the bipartite DNA organization of *Vibrionaceae* represents a special case because Chr 1 stretches from pole to pole, whereas Chr 2 prolongates from the new pole towards the cell center, thus suggesting that the chromatin density varies between the two halves of the cell. Higher chromatin density will presumably reduce the diffusion of macromolecular particles, such as proteins and ribosomes, in the nucleoid/DNA meshwork. Given that the DNA density is lower in the old pole area, the extra cytoplasmic space will presumably result in increased diffusion and transport of gene products, which provides a plausible explanation for the high abundance of core genes (many of which are growth related), and also the ribosomal protein clusters and rRNA clusters, in this subcellular region. Production of core gene products will therefore coincide and co-localize with the greatest number of growth/survival-related reactions and processes in the cell. A number of such cases can be mentioned, albeit we highlight two potential cases below.

The insertion of peptidoglycan (PG) in the cell wall happens in a dispersed manner, with the active growth zones along the axis [54]. To form the inner curvature of *Vibrio* cells, PG insertion is biased along the outer curve. Genes involved in cell wall synthesis are located in close proximity to *ori1* on *V. cholerae* Chr 1, with the main gene cluster related to nascent PG synthesis positioned approximately 0.38 Mb from *ori1.* This suggests that the first step of PG synthesis preferentially takes place in the old pole area. Similarly, motility related genes are found clustered 0.6 Mb from *ori1*, which is spatially close to the flagellum at the old pole.

## Conclusions

Our results show a non-random organization of pangene categories on the two chromosomes of *Vibrionaceae*, with an overrepresentation of core and softcore genes around *ori1.* Gene distribution was compared with global gene expression trends and showed that during fast growth, all pangene categories contribute to a skewed expression pattern in respect to *ori1*. From our data and previous literature, we can deduce that core and softcore genes are overrepresented at the old pole area of *V. cholerae*. We hypothesize that this pattern can be beneficial due to spatial links between the structural organization of core genes and their cellular function, and that differences in intracellular DNA densities might further contribute to the biased gene distribution. These findings add to the growing list of examples of spatial order in bacteria, and scientists will surely continue to study the interplay between genome organization, gene activity and cellular function. We envision to explore how different pangene categories are distributed on chromosomes of other bacterial orders, and to search for similar spatial links to gene functions to investigate if our current findings are part of a general trend in Bacteria, or specific to *Vibrionaceae*.

## Methods

### Genome retrieval and gene annotation

As of May 2018 a total of 124 complete *Vibrionaceae* genomes were publicly available at the National Center for Biotechnology Information (NCBI) which were downloaded from the RefSeq database at NCBI [55] (see Additional file 1 for a complete list). All genome sequences were re-annotated using RAST (Rapid Annotation using Subsystem Technology) version 2.0 [56] with default settings. The annotation of the 124 genome sequences resulted in a total of 555,513 annotated protein sequences.

### Pangenome approach to extract core, softcore, shell and cloud genes from large genome dataset

To categorize the annotated *Vibrionaceae* protein sequences into four categories (core, softcore, shell and cloud genes) we performed pangenome analysis using the software package GET_HOMOLOGUES (v3.1.0 (20180103)) [23]. The clustering algorithm OrthoMCL was used to cluster homolog protein sequences. The parameter “minimum percent sequence identity” was set to 50 and “minimum percent coverage in BLAST query/subj pairs” was set to 75 (default).

### Comparison of core, softcore, shell and cloud genes from 11 species

We chose 11 representative species (based on phylogeny and scientific interest i. e. number of papers published in PubMed) to study the distribution of core, softcore, shell and cloud genes on Chr 1 and Chr 2. Chr 1 and Chr 2 were divided into “upper half” (close to *ori*) and “lower half” (close to *ter*) and the number of core, softcore, shell and cloud genes in each half were counted (see Additional file 3). The 11 species were used to study the exact chromosomal positions of core and shell genes on Chr 1 and Chr 2. The DoriC database [57] was used to locate *ori1* and *ori2* in Chr 1 and Chr 2 to subsequently center the plotted chromosomes at origin of replication, respectively at *mioC* on Chr 1 and *rtcB* on Chr 2. The software package Circos [24] was used to visualize the gene distributions on the chromosomes.

### Analysing gene expression: mapping of read files on reference genomes

To study gene expression of core, softcore, shell and cloud genes in *A. salmonicida* LFI1238 and *V. natriegens* ATCC 14048 (NBRC 15636, DSM 759), the following datasets were downloaded from the Sequence Read Archive [28] at the NCBI: for *V. natriegens* ATCC 14048 datasets from growth in minimal (BioSample accession no. SAMN10926309, SAMN10926310 and SAMN10926313) and optimal (rich) medium (sample no. SAMN10926311, SAMN10926312 and SAMN10926329) at 37 °C to OD_600nm_ 0.3—0.5 [29]; for *A. salmonicida* LFI1238 one dataset (sample no. SAMEA4548122, SAMEA4548133, SAMEA4548134) originating from growth in LB medium containing 1% NaCl at 8 °C to mid log phase (OD_600nm_ ~ 0.5) [30]. The salt concentration is expected to be similar to the concentration the bacterium would experience inside its natural host (Atlantic salmon), where the bacterium is known to cause cold water vibriosis at temperatures below 10 °C [26, 27]. Hence, 8 °C was used in the experiment. The quality of the reads was checked using FastQC [58]. EDGE-pro v1.0.1 (Estimated Degree of Gene Expression in Prokaryotes) [31] in Galaxy was used to align cDNA reads to *V. natriegens* ATCC 14048 (assembly no. GCA_001456255.1) and *A. salmonicida* LFI1238 (assembly no. GCF_000196495.1) and estimate gene expression as reads per kilobase per million (RPKM) for all protein coding sequences (CDS). The RPKM values were then used to calculate the log_2_ ratio RPKM CDS:RPKM median to make global expression maps for each of the three datasets.

### Statistical analysis

Statistical analysis was performed using R in RStudio. Significance of gene distribution on either the upper or lower half of the chromosomes was performed using R’s chisq.test() function for the non-parameteric chi-squared test (see Additional file 4). Significance of gene expression between gene classes located on the upper or lower half of the chromosomes was performed using R’s wilcox.test() function for unpaired Wilcoxon signed-rank tests (see Additional file 4). For both analyses *P*-values were Bonferroni corrected for multiple comparisons using R’s p.adjust() function.

## Supplementary information

**Additional file 1: Table S1.** Complete list of the 124 *Vibrionaceae* genomes used in this study.

**Additional file 2: Fig. S1.** The 11 *Vibrionaceae* representatives mapped to a phylogeny.

**Additional file 3: Table S2.** Distribution and percent of total number of CDSs per chromosome of core, softcore, shell and cloud genes on «upper half» and «lower half» of Chr 1 and Chr 2 of 11 representative *Vibrionaceae* genomes.

**Additional file 4: Table S3.** Statistical analysis of gene distribution (chi-squared test) and gene expression (Wilcoxon signed-rank test) between “upper half” and “lower half” of Chr 1 and Chr 2.

**Additional file 5: Fig. S2.** Global expression maps of *V. natriegens* ATCC 14048 (grown under slow-growing conditions) chromosomal genes centered around the median. Data points (log_2_ ratio RPKM CDS:RPKM median) for each CDS are shown, as well as a trend line averaged over a sliding window of 200 data points.

**Additional file 6: Fig. S3.** Circular visualization of pangene distribution and gene expression (log_2_ ratio RPKM CDS:RPKM median) of (a) *A. salmonicida* LFI1238 and *V. natriegens* ATCC 14048 grown under (b) fast- and (c) slow-growing conditions.

## Data Availability

All data analysed during this study are included in this published article, its additional files and publicly available repositories. The RNA-seq datasets used in this study are available at Sequence Read Archive at Bioproject Accession PRJNA522293 [29] and PRJEB17700 [30].
