# Supplementary material for: Vibrionaceae core, shell and cloud genes are non-randomly distributed on Chr 1: An hypothesis that links the genomic location of genes with their intracellular placement
Source: BMC Genomics. 2020 Oct 6;21:695. doi: 10.1186/s12864-020-07117-5 (PMC7542380; doi:10.1186/s12864-020-07117-5)
Supplement: Supplementary file 2 — Additional file 2: Fig. S1. The 11 Vibrionaceae representatives mapped to a phylogeny. [file 12864_2020_7117_MOESM2_ESM.pdf]

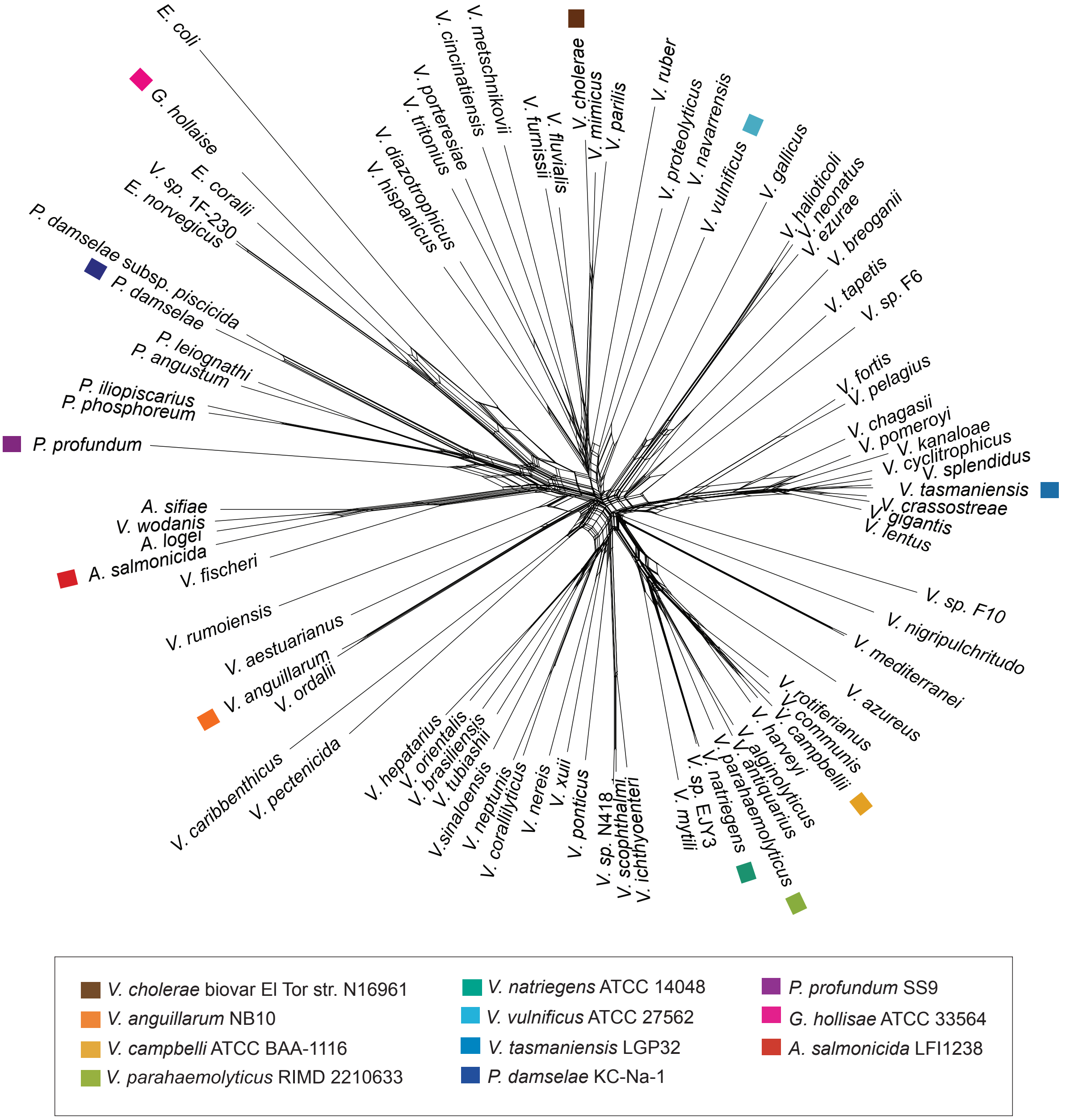

Figure S1:
The 11 representative *Vibrionaceae* genomes mapped to a phylogeny. The phylogenetic split network is based on a dataset from Sawabe and co-workers (Sawabe et al 2013. Frontiers in Microbiology 4(414). DOI:10.3389/fmicb.2013.00414) and consists of concatenated genes *ftsZ, gap, gyrB, pyrH, recA, rpoA* and *topA*. The tree was constructed using SplitsTree4 to concatenate the individual gene alignments and settings for network were uncorrected P and NeighborNet.
