# Supplementary material for: Vibrionaceae core, shell and cloud genes are non-randomly distributed on Chr 1: An hypothesis that links the genomic location of genes with their intracellular placement
Source: BMC Genomics. 2020 Oct 6;21:695. doi: 10.1186/s12864-020-07117-5 (PMC7542380; doi:10.1186/s12864-020-07117-5)
Supplement: Supplementary file 5 — Additional file 5: Fig. S2. Global expression maps of V. natriegens ATCC 14048 (grown under slow-growing conditions) chromosomal genes centered around the median. Data points (log2 ratio RPKM CDS:RPKM median) for each CDS are shown, as well as a trend line averaged over a sliding window of 200 data points. [file 12864_2020_7117_MOESM5_ESM.pdf]

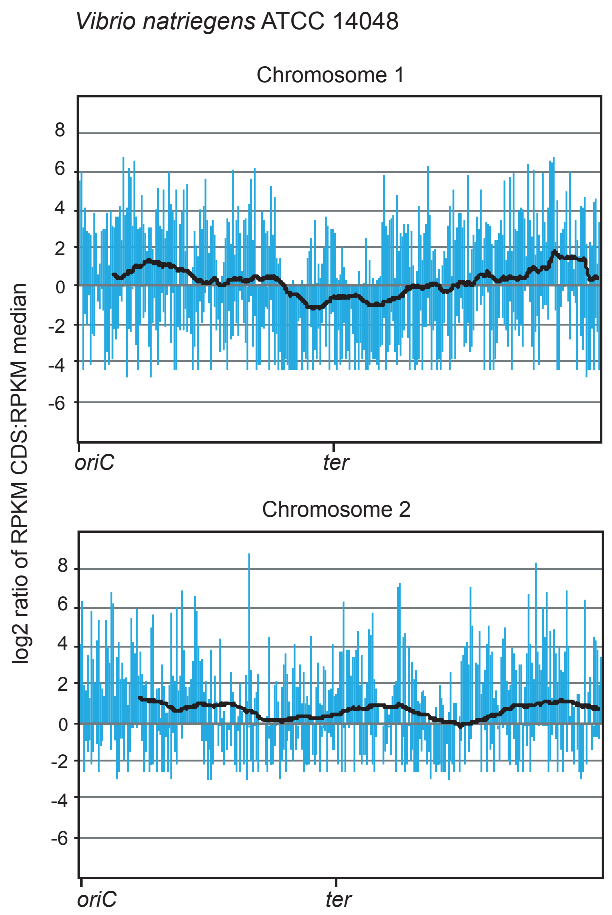

Figure S3:
Global expression maps of *V. natriegens* ATCC 14048 (grown under slow-growing conditions) chromosomal genes centered around the median. Data points (log_2 ratio RPKM CDS:RPKM median) for each CDS are shown, as well as a trend line averaged over a sliding window of 200 data points.
