# Supplementary material for: Vibrionaceae core, shell and cloud genes are non-randomly distributed on Chr 1: An hypothesis that links the genomic location of genes with their intracellular placement
Source: BMC Genomics. 2020 Oct 6;21:695. doi: 10.1186/s12864-020-07117-5 (PMC7542380; doi:10.1186/s12864-020-07117-5)
Supplement: Supplementary file 6 — Additional file 6: Fig. S3. Circular visualization of pangene distribution and gene expression (log2 ratio RPKM CDS:RPKM median) of (a) A. salmonicida LFI1238 and V. natriegens ATCC 14048 grown under (b) fast- and (c) slow-growing conditions. [file 12864_2020_7117_MOESM6_ESM.pdf]

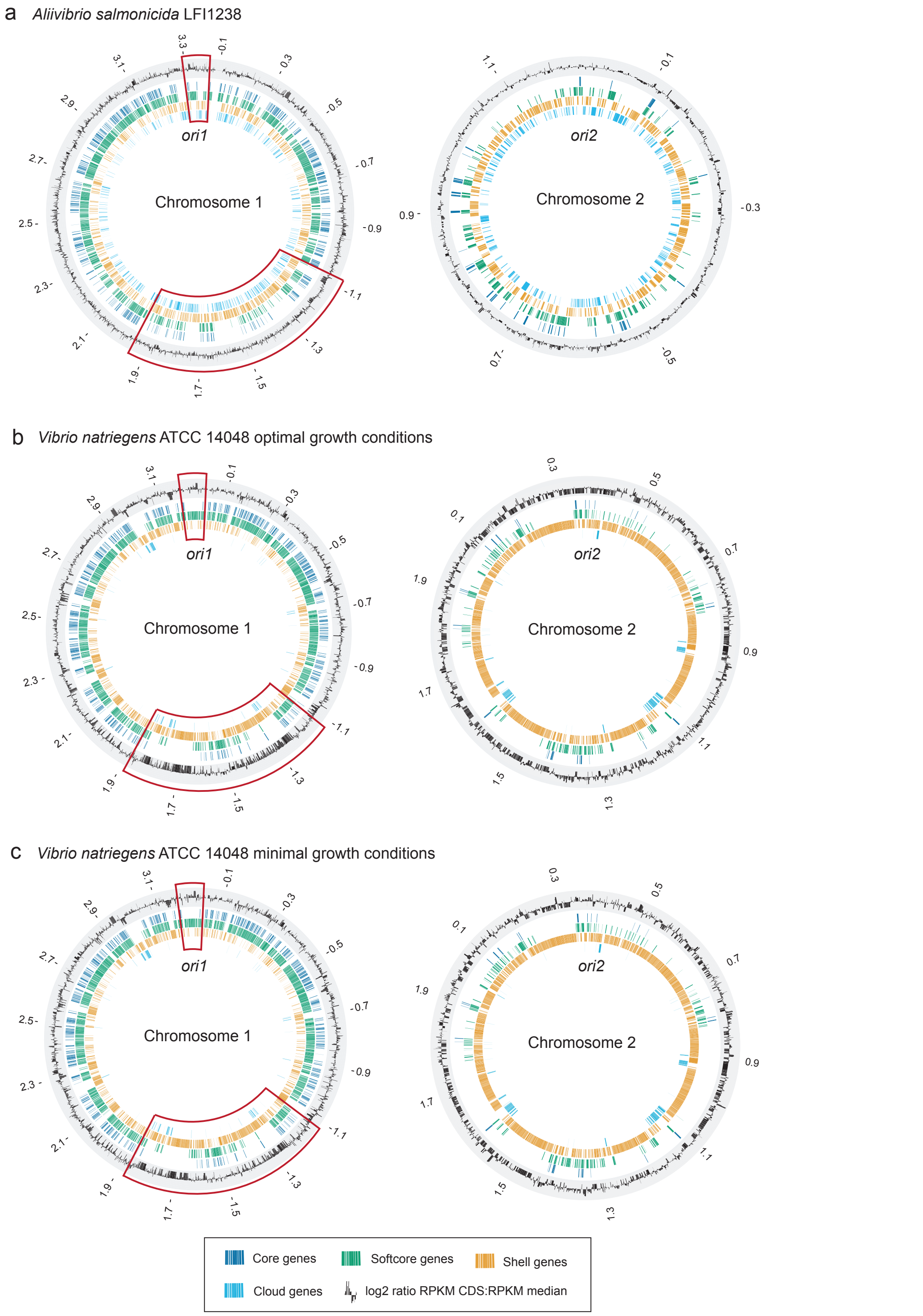


**Figure S2:**
Circular visualization map of all pangene categoires and gene expression patterns ($log_2$ ratio RPKM CDS:RPKM median) of (a) *A. salmonicida* LFI1238 and *V. natriegens* ATCC 14048 grown under (b) fast- and (c) slow-growing conditions. The areas marked in red correspond to "i" and "ii" in Fig. 2. Megabases are labeled as ticks. The genome comparison tool Circos [24] was used to create the figure.
